# Cancer risk following surgical removal of tonsils and adenoids — a population-based, sibling-controlled cohort study in Sweden

**DOI:** 10.1186/s12916-023-02902-x

**Published:** 2023-05-24

**Authors:** Jinfeng Liang, Yi Huang, Li Yin, Fatemeh Sadeghi, Yanping Yang, Xue Xiao, Hans-Olov Adami, Weimin Ye, Zhe Zhang, Fang Fang

**Affiliations:** 1 Department of Otolaryngology-Head & Neck Surgery, First Affiliated Hospital of Guangxi Medical University, 22# Shuangyong Road, Guangxi 530021 Nanning, China; 2 Key Laboratory of Early Prevention and Treatment for Regional High-Frequency Tumor (Guangxi Medical University), Ministry of Education, Guangxi Key Laboratory of Early Prevention and Treatment for Regional High Frequency Tumor, Guangxi Medical University, Nanning, Guangxi, China; 3 Department of Medical Epidemiology and Biostatistics, Karolinska Institutet, Stockholm, Sweden; 4 Institute of Environmental Medicine, Karolinska Institutet, Stockholm, Sweden; 5 Clinical Effectiveness Group, Institute of Health and Society, University of Oslo, Oslo, Norway

**Keywords:** Tonsillectomy, Adenoidectomy, Adenotonsillectomy, Cancer, Risk, Sibling comparison

## Abstract

**Background:**

Removal of tonsils and adenoids is among the most common surgical procedures worldwide. Evidence of increased risk of cancer following such surgery is, however, inconclusive.

**Methods:**

We conducted a population-based, sibling-controlled cohort study of 4,953,583 individuals in Sweden with a follow-up during 1980–2016. History of tonsillectomy, adenotonsillectomy, and adenoidectomy was identified from the Swedish Patient Register whereas incident cases of cancer during follow-up were identified from the Swedish Cancer Register. We used Cox models to calculate hazard ratios (HR) with 95% confidence intervals (CI) of cancer in both a population and a sibling comparison. The sibling comparison was used to assess the potential impact of familial confounding, due to shared genetic or non-genetic factors within a family.

**Results:**

We found a modestly increased risk for any cancer following tonsillectomy, adenoidectomy, or adenotonsillectomy in both the population (HR 1.10; 95%CI 1.07–1.12) and sibling (HR 1.15; 95%CI 1.10–1.20) comparisons. The association did not differ greatly by type of surgery, age at surgery, or potential indication for surgery, and persisted more than two decades after surgery. An excess risk was consistently observed for cancer of the breast, prostate, thyroid, and for lymphoma in both population and sibling comparisons. A positive association was observed for pancreatic cancer, kidney cancer, and leukemia in the population comparison whereas a positive association was observed for esophageal cancer in the sibling comparison.

**Conclusions:**

Surgical removal of tonsils and adenoids is associated with a modestly increased risk of cancer during the decades following the surgery. The association is unlikely attributed to confounding due to shared genetic or non-genetic factors with a family.

**Supplementary Information:**

The online version contains supplementary material available at 10.1186/s12916-023-02902-x.

## Background

Tonsils and adenoids are secondary lymphoid organs with humoral and cellular immune functions in response to inhaled or ingested antigens [1]. Surgical removal of tonsils and adenoids, i.e., tonsillectomy, adenotonsillectomy, and adenoidectomy, is a common procedure used in the treatment of hypertrophic tonsils, chronic infection, obstructive sleep apnea, and recurrent middle ear effusion [2]. For instance, it is estimated that about 289,000 children undergo tonsillectomy annually in the USA [3], and an increasing number of tonsillectomy is performed among children of young age [4]. Given their wide application and the immune function of the organs, it seems important to assess health outcomes following these procedures. Indeed, an increased risk of inflammatory bowel disease [5], acute myocardial infarction [6], as well as respiratory, allergic, and infectious diseases [7] has been reported among adults or children with prior surgical removal of tonsils and adenoids, compared to others.

Risk of cancer after a surgical removal of tonsils and adenoids has also been studied with conflicting results (see Table 1 for a summary [2, 8–48]). For instance, some studies found tonsillectomy to be associated with an increased risk of Hodgkin's disease [8, 20], lymphocytic leukemia [11], tongue cancer [2], breast cancer [38], and prostate cancer [41], whereas other studies found tonsillectomy to be associated with a decreased risk of Hodgkin's disease [16], pancreatic cancer [35], and tonsil cancer [33], or not associated with risk of acute leukemia [30] or head and neck cancer (excluding tonsil cancer) [31]. Because few studies examined all cancers, inconsistent results might be due to real differences between cancer types but also to differences in study design and random error due to the small sample size.

**Table 1:** Summary of existing studies on cancer risk in relation to tonsillectomy

Study, country	Study design	Sample size	Finding
**Hematopoietic malignancies**			
Vestergaard (2010) [8] Denmark	Cohort study	2.1 million person-years in individuals with tonsillectomy; 72 incident cases of HD	Among persons under 15 years of age: 1–4 years after tonsillectomy, RR = 3.9 (95%CI: 1.4–11) > 5 years after tonsillectomy, RR = 3.5 (95%CI: 1.4–8.5)
Liaw (1997) [9] Sweden	Cohort study	55,169 individuals with tonsillectomy; 20 incident cases of HD	> 1 year after tonsillectomy: Overall SIR = 1.4 (95%CI: 0.9–2.2) Age at tonsillectomy < 12: SIR = 4.1 (95%CI: 1.6–8.4) Age at tonsillectomy 12–19: SIR = 1.3 (95%CI: 0.6–2.5) Age at tonsillectomy ≥ 20: SIR = 0.7 (95%CI: 0.2–1.8)
Becker (2005) [10] Germany	Case–control study	710 cases (115 HD, 554 B-NHL, 35 T-NHL, and 6 others) and 710 controls	Overall: HD: OR = 1.32 (95%CI: 0.80–2.19) Follicular lymphoma: OR = 1.84 (95%CI: 1.15–2.93) CLL: OR = 1.42 (95%CI: 0.88–2.29) B-NHL: OR = 1.24 (95%CI: 0.96–1.61) T-NHL: OR = 0.86 (95%CI: 0.36–2.06) MM: OR = 0.78 (95%CI: 0.42–1.43) Age at tonsillectomy ≤ 6 years: HD: OR = 2.05 (95%CI: 0.72–5.84) Follicular lymphoma: OR = 4.93 (95%CI: 1.97–12.3) CLL: OR = 4.01 (95%CI: 1.43–11.3) B-NHL: OR = 2.97 (95%CI: 1.57–5.62) T-NHL: OR = 3.46 (95%CI: 0.69–17.3) MM: OR = 1.00 (95%CI: 0.13–8.12)
Vineis (2003) [11] Italy	Case–control study	574 cases (261 LL and 313 ML) and 1718 controls	LL: OR = 0.9 (95%CI: 0.7–1.3) ML: OR = 0.9 (95%CI: 0.6–1.1)
Vineis (2000) [12] Italy	Case–control study	2669 cases (1388 NHL, 354 HD, 263 MM, 261 LL, 313 ML, and 90 others) and 1718 controls	NHL: OR = 1.1 (95%CI: 0.9–1.3) HD: OR = 1.0 (95%CI: 0.7–1.3) MM: OR = 0.8 (95%CI: 0.6–1.2) LL: OR = 0.8 (95%CI: 0.6–1.1) ML: OR = 0.8 (95%CI: 0.6–1.1)
Schüz (1999) [13] Germany	Case–control study	1010 children with acute leukemia and 1010 controls	OR = 1.4 (95%CI: 1.0–1.9)
Gledović (1991) [14] Serbia	Case–control study	113 cases with HD, 113 neighborhood controls, and 113 hospital controls	Neighborhood controls: OR = 0.91 (95%CI: 0.45–1.81) Hospital controls: OR = 0.74 (95%CI: 0.38–1.43)
Serraino (1991) [15] Italy	Case–control study	152 cases with HD and 613 controls	Any histology type: OR = 0.9 (95%CI: 0.6–1.4) Nodular sclerosis: OR = 1.1 (95%CI: 0.6–1.9)
Bonelli (1990) [16] Italy	Case–control study	160 cases with HD and 185 hospital controls	Age at tonsillectomy < 10: RR = 0.46 (95%CI: 0.22–0.94) Age at tonsillectomy 11–20: RR = 1.10 (95%CI: 0.39–2.82) Age at tonsillectomy ≥ 21: RR = 1.14 (95%CI: 0.37–3.51)
Mueller (1987) [17] USA	Case–control studies	684 cases with HD and 786 sibling controls	15–39 years: RR = 1.0 (95%CI: 0.72–1.4) 40–54 years: RR = 1.5 (95%CI: 0.67–3.3) ≥ 55 years: RR = 3.0 (95%CI: 1.3–6.9)
Hardell (1983) [18] Sweden	Case‐control study	59 cases with HD and 117 controls	RR = 2.7 (95%CI: 0.6–11.6)
Silingardi (1982) [19] Italy	Case–control study	505 cases with HD, 226 cases with NHL, and 731 controls	HD: RR = 0.88 (*p*: NS) NHL: RR = 1.23 (*p*: NS)
Vianna (1980) [20] USA	Case‐control study	81 cases with HD and 81 sibling controls	RR = 2.7 (95%CI: 1.1–6.5)
Kirchhoff (1980) [21] Brazil	Case–control study	70 cases with HD and 128 sibling controls	RR = 2.5 (95%CI: 1.0–6.0)
Henderson (1979) [22] USA	Case–control study	218 cases with HD and 218 controls	RR = 0.98 (*p*: NS)
Andersen (1978) [23] Denmark	Case‐control study	63 cases with HD and 182 controls	No association (*p* = 0.79)
Abramson (1978) [24] Israel	Case–control study	403 of cases with HD and 403 controls	HD: RR = 1.3 (*p*: NS) Mixed cellularity: RR = 8.5 (*p* < 0.0004) Nodular sclerosis: RR = 0.7 (*p*: NS)
Paffenbarger (1977) [25] USA	Case–control study	45 cases with HD and 180 classmate controls	RR = 0.9 (*p*: 0.84)
Vianna (1974) [26] USA	Case–control study	95 cases with HD and 95 sibling controls	RR = 2.0 (95%CI: 1.1–3.6)
Johnson (1972) [27] USA	Case–control study	174 cases with HD and 472 sibling controls	No association (*p*: NS)
Vianna (1971) [28] USA	Case–control study	109 cases with HD and 109 controls	67 of 101 cases vs. 43 of 107 controls with tonsillectomy (*p* < 0.001)
Ruuskanen (1971) [29] Finland	Case–control study	53 cases with HD and 53 controls	5 of 53 cases vs. 7 of 53 controls with tonsillectomy (*p*: NS)
Freeman (1971) [30] USA	Case–control study	310 children with acute leukemia and 855 controls	No association (*p*: NS)
**Solid tumors**			
Chaturvedi (2016) [31] Sweden	Cohort study	225,718 individuals with tonsillectomy; 144 incident cases of head and neck cancer	> 1 year after tonsillectomy: Tonsil: SIR = 0.31 (95%CI: 0.08–0.79) Non-tonsil oropharyngeal: SIR = 1.61 (95%CI: 0.77–2.95) Base of tongue: SIR = 1.63 (95%CI: 0.66–3.36) Other head and neck: SIR = 0.92 (95%CI: 0.64–1.27) > 5 years after tonsillectomy: Tonsil: SIR = 0.17 (95%CI: 0.02–0.62) Base of tongue: SIR = 1.31 (95%CI: 0.42–3.05)
Fakhry (2015) [32] Denmark	Cohort study	90,775 individuals with tonsillectomy; 52 incident cases of oropharyngeal cancer	> 1 year after tonsillectomy: < 60 years: Oropharyngeal: RR = 0.6 (95%CI: 0.4–0.9) Tonsil: RR = 0.2 (95%CI: 0.06–0.4) Base of tongue: RR = 0.5 (95%CI: 0.2–1.4) ≥ 60 years: Oropharyngeal: RR = 2.5 (95%CI: 1.7–3.8) Tonsil: RR = 1.8 (95%CI: 0.9–3.6) Base of tongue: RR = 4.2 (95%CI: 1.8–9.3)
Combes (2021) [33] France	Case-case study	363 cases with oropharyngeal cancer and 682 cases with non-oropharyngeal head and neck cancers	Oropharyngeal: OR = 1.1 (95%CI: 0.8–1.4) Tonsil: OR = 0.4 (95%CI: 0.2–0.8) Base of tongue: OR = 1.8 (95%CI: 1.1–3.1) Tonsil and base of tongue: OR = 0.9 (95%CI: 0.6–1.4) Other oropharyngeal cancer: OR = 1.2 (95%CI: 0.9–1.7)
Garman (2020) [34] USA	Case–control study	396 cases with esophageal adenocarcinoma and 1102 controls	OR = 1.8 (95%CI: 1.2–2.7)
Zevallos (2016) [2] USA	Case–control study	361 cases with oropharyngeal cancer and 1378 controls	Oropharyngeal: OR = 0.63 (95%CI: 0.47–0.85) Base of tongue: OR = 1.95 (95%CI: 1.25–3.06) HPV-positive base of tongue: OR = 2.46 (95%CI: 1.22–4.95) Tonsil: OR = 0.22 (95%CI: 0.13–0.36) HPV-positive tonsil: OR = 0.17 (95%CI: 0.08–0.34) P16-positive base of tongue: OR = 2.24 (95%CI: 1.16–4.35) P16-positive tonsil: OR = 0.14 (95%CI: 0.07–0.31)
Zhang (2014) [35] USA	Case–control study	215 cases with pancreatic cancer and 676 controls	OR = 0.67 (95%CI: 0.48–0.94)
Brasky (2009) [36] USA	Case–control study	740 cases with breast cancer and 810 controls	Premenopausal: OR = 1.50 (95%CI: 1.08–2.08) Postmenopausal: OR = 1.05 (95%CI: 0.79–1.38)
Zivaljevic (2004) [37] Serbia	Case–control study	110 cases with thyroid cancer and 110 hospital controls	OR = 1.11 (95%CI: 0.58–2.11)
Yasui (2001) [38] USA	Case–control study	537 cases with breast cancer and 492 controls	Age at tonsillectomy 0–4: OR = 0.82 (95%CI: 0.50–1.35) Age at tonsillectomy 5–9: OR = 1.05 (95%CI: 0.76–1.43) Age at tonsillectomy 10–14: OR = 1.08 (95%CI: 0.72–1.64) Age at tonsillectomy ≥ 15: OR = 1.68 (95%CI: 1.09–2.6)
Ilić (1996) [39] Serbia	Case–control study	101 cases with prostate cancer and 202 controls	7 of 101 cases vs. 10 of 202 controls with tonsillectomy (*p*: NS)
Bueno (1992) [40] Netherlands	Case–control study	176 cases with exocrine pancreatic carcinoma and 487 controls	OR = 0.86 (95%CI: 0.55–1.36)
Whittemore (1985) [41] USA	Case–control study	243 cases with prostate cancer and 972 controls	RR = 1.9 (95%CI: 1.4–2.6)
Gold (1985) [42] USA	Case–control study	201 cases with pancreatic cancer and 402 controls (201 hospital controls and 201 non-hospital controls)	Hospital controls: OR = 0.29 (95%CI: 0.14–0.59) Non-hospital controls: OR = 0.26 (95%CI: 0.12–0.59)
Lubin (1982) [43] Canada	Case–control study	577 cases with breast cancer and 826 controls	> 65 years: RR = 2.3 (95%CI: 1.3–3.9)
**Any cancer**			
Sun (2015) [44] Taiwan	Cohort study	997 individuals with tonsillectomy, 37 incident cases of cancer	> 3 years after tonsillectomy: IRR = 1.57 (95%CI: 1.02–2.41)
Cassimos (1973) [45] Greece	Case–control study	500 patients with cancer and 500 controls	11 of 500 cases vs. 45 of 500 controls with tonsillectomy (*p* < 0.01)
Gross (1966) [46] USA	Case–control study	300 cases with cancer and 200 controls with noncancerous diseases	23% of cases vs. 24% of controls with tonsillectomy (*p*: NS)
Howie (1996) [47] Scotland	Case–control study	1019 cases with cancer and 623 controls	23% of cases vs. 12% of controls with tonsillectomy (*p* < 0.05)
Kessler (1969) [48] USA	Case–control study	461 cases with cancer and 223 controls	Overall: RR = 1.17 (*p*: NS) Male: RR = 1.26 (*p*: NS) Female: RR = 1.06 (*p*: NS) Cancer site: Buccal cavity (male): RR = 1.71 (*p*: NS) Colon and rectum (Male): RR = 1.87 (*p*: NS) Respiratory (male): RR = 1.43 (*p*: NS) Breast (female): RR = 1.07 (*p*: NS) Genitalia (female): RR = 0.79 (*p*: NS)

*RR* relative risk/risk ratio, *CI* confidence interval, *OR*, odds ratio, *SIR* standardized incidence rate ratio, *IRR* incidence rate ratio, *HD* Hodgkin’s disease, *NHL* non-Hodgkin’s lymphoma, *B-NHL* B-cell non-Hodgkin lymphoma, *MM* multiple myeloma, *LL* lymphocytic leukemia, *ML* myeloid leukemia, *NS* not statistically significant, *NA* not available

To this end, we performed a large population-based and sibling-controlled cohort study in Sweden to assess the long-term risk of the entire spectrum of malignancies following tonsillectomy, adenotonsillectomy, or adenoidectomy in either childhood or adulthood. The sibling-controlled design was used to reduce familial confounding, due to shared genetic or non-genetic risk factors within a family.

## Methods

### Study design

We performed a cohort study including all individuals born during 1932–2016 in Sweden, whose parents were also born in Sweden, according to the Swedish Total Population Register. We followed these individuals from January 1st, 1980, or date of birth, whichever came later, to a first diagnosis of cancer, emigration out of Sweden, death, or December 31st, 2016, whichever came first, through cross-linkages to the Swedish Cancer Register, Total Population Register, and Causes of Death Register, using the individually unique personal identification numbers in Sweden. If an individual had a cancer diagnosis, emigrated out of Sweden, or died before cohort entry, we excluded them from the cohort. The final cohort included 4,953,583 individuals. We identified all newly diagnosed cancers during the follow-up of the cohort from the Swedish Cancer Register, which has since 1958 collected information on all incident malignancies in Sweden [49]. We studied first the risk of any cancer and then by specific sites and types of cancer. Additional File 1: Table S1 shows the diagnostic codes used to define different cancer types.

We identified tonsillectomy, adenotonsillectomy, and adenoidectomy from the Swedish Patient Register, which has since 1964 recorded information on hospital-based inpatient care in Sweden. The Register records admission and discharge dates, primary and secondary diagnoses, as well as surgical procedures [50]. Although the Register has nationwide coverage for inpatient care since 1987, it covered more than 80% of all inpatient care in the country since 1980. From 2001 onward, the Register has also included more than 80% of hospital-based outpatient care records in Sweden. We used surgical codes 2710 before 1997 and EMB10 since 1997 for tonsillectomy, 2720 before 1997 and EMB20 since 1997 for adenotonsillectomy, and 2720 and 2730 before 1997 and EMB30 since 1997 for adenoidectomy. We also used EMB99 since 1997 to define other resection or excision of tonsils or adenoids, but there was no corresponding code before 1997. As these procedures have been exclusively performed during inpatient care until 2006 in Sweden [31], we identified the surgical codes from both inpatient and outpatient care.

The analytical cohort included in total 589,229 individuals with recorded surgical removal of tonsils and adenoids (11.9%) and 4,364,354 individuals without such exposure (88.1%), comprising the population comparison. We treated the exposure status as a time-varying variable. Hence, if an individual had such a procedure before cohort entry, they contributed all follow-up time to the exposed group. If an individual had such a procedure during follow-up, they contributed follow-up time to the unexposed group before the surgery and to the exposed group after the surgery. If an individual had no such surgery either before or during follow-up, they contributed all person-time to the unexposed group.

In addition to the population comparison, we also performed a sibling comparison where we included 107,910 participants of the cohort with recorded surgical removal of tonsils and adenoids who had at least one full sibling together with 186,093 unexposed full siblings of these individuals. We used the sibling comparison to reduce the potential impact of familial confounding, due to genetic and non-genetic factors shared between full siblings, on the studied association.

### Statistical analyses

We first summarized for both the population and sibling comparisons characteristics of the study participants, including sex, age at cohort entry, and educational attainment by exposure status. We obtained information on the highest educational attainment during the study period (i.e., 0–9 years, 10–12 years, ≥ 13 years, or “unknown”), as a proxy for socioeconomic status, for all participants through the Swedish Longitudinal Integrated Database for Health Insurance and Labor, which has since 1990 collected annually updated information on demographic and socioeconomic status for individuals above 16 years in Sweden. We then calculated the crude incidence rate (IR) of cancer by exposure status, dividing the number of incident cancer cases by the accumulated person-years. Finally, in the population comparison, we used Cox models to estimate the average hazard ratio (HR) and 95% confidence interval (CI) of cancer in relation to the exposure. We used attained age as the underlying time scale and adjusted for sex, educational attainment, and calendar period during follow-up. In the sibling comparison, we used conditional Cox models with family identifiers as the strata and the same adjustment as in the population comparison. To alleviate concern on surveillance bias (i.e., individuals who underwent a surgical procedure might be more surveyed immediately following the procedure, compared to others, leading to a higher-than-expected rate of cancer detection) or reverse causation (i.e., a surgical procedure might be given for a condition that is secondary to an upcoming cancer), we excluded in all analyses the first three years of follow-up from the exposed group and included instead these years in the unexposed group.

We first analyzed any cancer as one outcome and then separately by site and type of cancer. We then focused on any cancer as outcome and performed analyses by type of surgery (i.e., tonsillectomy, adenotonsillectomy, or adenoidectomy), age at surgery, potential indication for surgery, and time since surgery, to examine whether the risk of cancer would differ by type of surgery, between young and older age at surgery, or between individuals with or without documented indications, as well as to understand the temporal pattern of cancer risk following such surgery. For the exposed group, we identified from the Swedish Patient Register any hospital visit with a discharge diagnosis of hypertrophy of tonsils and adenoids, chronic infection in tonsils and adenoids, sleep-related conditions, and other chronic diseases of the tonsils and adenoids before the date of surgery and considered them as potential indications for surgery. Additional File 1: Table S2 shows the diagnostic codes used to define these indications. To assess potential effect modifiers, we also performed stratified analyses by sex, age at follow-up, and educational attainment for any cancer. Specifically, we also examined the associations for individual sites and types of cancer among men and women. Assuming the normal distribution for the estimated HRs, we used Chi-square test to examine the statistical significance for the difference or a potential trend between the HRs of different subgroups.

Finally, we performed a series of sensitivity analyses based on the population comparison to assess the robustness of the findings. First, to understand the potential influence of indication bias, namely that it is the indications for the surgical removal of tonsils or adenoids, rather than the procedure itself, which are associated with the risk of cancer, we performed a sensitivity analysis comparing the exposed individuals with a potential indication for the surgery (i.e., with a discharge diagnosis of hypertrophy of tonsils and adenoids, chronic infection in tonsils and adenoids, sleep-related conditions, and other chronic diseases of the tonsils and adenoids) to the unexposed individuals with a discharge diagnosis of these conditions without however experiencing the procedure (“indication group”). Second, some individuals had the procedure before 1980 and had to be free of cancer until cohort entry to be included in the analysis. In a second sensitivity analysis, we excluded these individuals from the exposed group to assess the potential influence of such “survival bias” and to assess if such bias varied by age. Finally, we in the main analysis excluded the first three years of follow-up from the exposed group and included them in the unexposed group. However, the risk of cancer during the immediate time following the surgical removal is also interesting to estimate. We therefore performed a third sensitivity analysis including the first three years of follow-up in the exposed group instead.

Data management and analyses were performed using SAS version 9.4 (SAS Institute Inc, Cary, NC) and R version 3.6.0. A two-sided *P* < 0.05 was considered statistically significant. We did not adjust for multiplicity of statistical tests, as adopting a top-down approach, the main hypothesis of increased risk of cancer in relation to surgical removal of tonsils and adenoids consists of only one test.

## Results

Individuals with a surgical removal of tonsils or adenoids were more likely female and younger at cohort entry, and had higher educational attainment, compared with others, in both the population and sibling comparisons (Table 2).

**Table 2:** Baseline characteristics of study participants by status of surgical removal for tonsils and adenoids, a population-based and sibling-controlled cohort study during 1980–2016 in Sweden

	**Population comparison**		**Sibling comparison**	
**Characteristics**	**Individuals with surgery**	**Unexposed population reference**	**Individuals with surgery**	**Unaffected siblings**
**Sex**				
Male	67,345 (43.5%)	2,416,969 (51.6%)	46,875 (43.4%)	96,653 (51.9%)
Female	87,425 (56.5%)	2,268,040 (48.4%)	61,035 (56.6%)	89,440 (48.1%)
**Age at cohort entry**				
0–6	38,796 (25.1%)	635,775 (13.6%)	25,877 (24.0%)	38,118 (20.5%)
7–12	30,905 (20.0%)	603,560 (12.9%)	21,509 (19.9%)	32,449 (17.4%)
13–18	28,928 (18.7%)	626,305 (13.4%)	20,688 (19.2%)	32,609 (17.5%)
19–45	55,259 (35.7%)	2,620,209 (55.9%)	39,557 (36.7%)	81,667 (43.9%)
46 +	882 (0.6%)	199,160 (4.4%)	279 (0.3%)	1250 (0.7%)
**Educational attainment**				
–9 years	20,358 (13.2%)	961,895 (20.5%)	13,922 (12.9%)	32,134 (17.3%)
> 9–12 years	76,393 (49.4%)	2,085,097 (44.5%)	52,677 (48.8%)	89,674 (48.2%)
> 12 years	56,965 (36.8%)	1,550,080 (33.1%)	40,860 (37.9%)	63,012 (33.9%)
Unknown	1054 (0.7%)	87,937 (1.9%)	451 (0.4%)	1273 (0.7%)

**Fig. 1:**
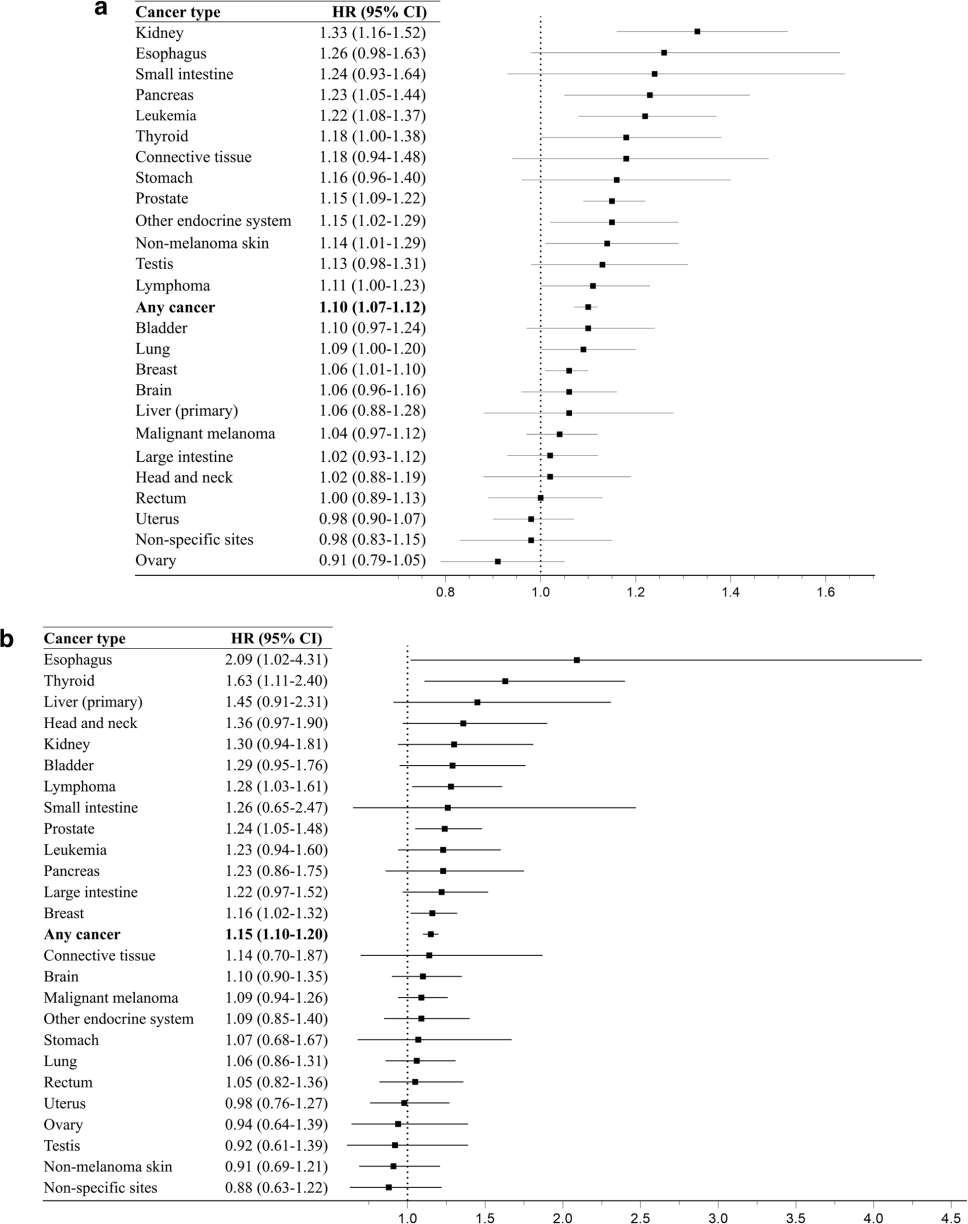
Hazard ratio (HR) and 95% confidence interval (CI) for cancer in relation to a surgical removal of tonsils or adenoids, results of any cancer and by site and group of cancer in the population (**a**) or sibling (**b**) comparison

In the population comparison, individuals with a surgical removal of tonsils or adenoids had a slightly higher risk of any cancer (HR 1.10; 95%CI 1.07–1.12), compared with individuals without such surgery, after adjustment for sex, age, and calendar period at follow-up, and educational attainment (Fig. 1a, Additional File 1: Table S3). A statistically significant positive association was noted for cancer of the pancreas (HR 1.23; 95%CI 1.05–1.44), breast (HR 1.06; 95%CI 1.01–1.10), prostate (HR 1.15; 95%CI 1.09–1.22), kidney (HR 1.33; 95%CI 1.16–1.52), and thyroid (HR 1.18; 95%CI 1.00–1.38), as well as non-melanoma skin cancer (HR 1.14; 95%CI 1.01–1.29), other cancer in the endocrine system (HR 1.15; 95%CI 1.02–1.29), lymphoma (HR 1.11; 95%CI 1.00–1.23), and leukemia (HR 1.22; 95%CI 1.08–1.37). The association for lymphoma was mainly noted for non-Hodgkin lymphoma (HR 1.15; 95%CI 1.03–1.29). Although not statistically significant, a positive association was also suggested for esophageal cancer.

In the sibling comparison, we also found an increased risk of any cancer following the procedure (HR 1.15; 95%CI 1.10–1.20) (Fig. 1b, Additional File 1: Table S4). A positive association was noted for cancer of the esophagus (HR 2.09; 95%CI 1.02–4.31), breast (HR 1.16; 95%CI 1.02–1.32), prostate (HR 1.24; 95%CI 1.05–1.48), thyroid (HR 1.63; 95%CI 1.11–2.40), and for lymphoma (HR 1.28; 95%CI 1.03–1.61). Although not statistically significant, a positive association was also suggested for pancreatic cancer, kidney cancer, and leukemia.

The increased risk of any cancer did not vary greatly by type of surgery, age at surgery, or potential indication for surgery in either the population or sibling comparison (Table 3). The association persisted more than 20 years after surgery. The HR was 1.14 (95%CI 1.07–1.23) during 11–20 years after surgery and 1.09 (95%CI 1.06–1.21) from 21 years onward after surgery in the population comparison and 1.19 (95%CI 1.01–1.41) during 11–20 years after surgery and 1.12 (95%CI 1.06–1.18) from 21 years onward after surgery in the sibling comparison. Finally, the stratified analyses by sex, age at follow-up, and educational attainment did not disclose any statistically significant interactions between the procedure and these variables (Table 4). The stratified analyses by sex, age at follow-up, and educational attainment did not disclose any statistically significant interactions between the procedure and these variables (Table 4). Finally, the associations for individual sites and types of cancer did not differ greatly between men and women either (Additional File 1: Table S5).

**Table 3:** Incidence rate (IR, per 100,000 person-years) and hazard ratio (HR) with 95% confidence interval (CI) of any cancer in relation to surgical removal of tonsils and adenoids, analysis by characteristics of the surgery, a population-based and sibling-controlled cohort study (3-year lag time)*

	**Population comparison**		**Sibling comparison**	
**Characteristics**	***N*** **of cases/IR**	**HR (95%CI)**^**a**^	***N*** **of cases/IR**	**HR (95%CI)**^**a**^
**Type of surgery**				
No surgery	579,451/358.0	Ref	17,735/228.2	Ref
Tonsillectomy	8575/296.0	1.09 (1.07–1.11)	6016/292.6	1.14 (1.09–1.20)
Adenotonsillectomy	409/155.9	1.17 (1.05–1.28)	308/163.3	1.05 (0.85–1.31)
Adenoidectomy	890/138.3	1.10 (1.03–1.17)	662/143.7	1.15 (0.99–1.33)
*P for difference*		0.28		0.71
**Age at surgery**				
No surgery	579,451/358.0	Ref	17,735/228.2	Ref
1–6	30/56.1	1.13 (0.79–1.62)	18/56.2	0.96 (0.29–3.12)
7–12	302/72.8	1.13 (1.00–1.29)	209/77.5	1.50 (0.94–2.40)
13–18	777/112.1	1.02 (0.94–1.11)	545/115.4	1.21 (0.92–1.58)
19–45	7501/305.5	1.08 (1.05–1.10)	5316/302.6	1.12 (1.06–1.19)
46–	1168/1114.6	1.07 (0.97–1.17)	760/1102.3	1.06 (0.61–1.84)
*P for difference*		0.63		0.57
**Potential indications for surgery**				
No surgery	579,451/358.0	Ref	17,735/228.2	Ref
Hypertrophy of tonsils and adenoids	5512/242.6	1.08 (1.05–1.12)	3853/245.2	1.19 (1.11–1.17)
Chronic tonsilitis, pharyngitis and nasopharyngitis, or peritonsillar abscess	2718/255.7	1.09 (1.07–1.12)	1947/255.8	1.12 (1.06–1.18)
Sleep disorders or dyspnea and respiratory abnormalities	78/600.8	1.09 (1.07–1.12)	58/629.6	1.14 (1.09–1.20)
Other diseases of tonsils and adenoids	173/113.3	1.10 (1.07–1.12)	121/152.2	1.15 (1.10–1.20)
*P for difference*		0.74		0.36
**Time since surgery**				
No surgery	579,451/358.0	Ref	17,735/228.2	Ref
> 3–10 years	994/100.0	1.06 (0.92–1.21)	676/97.1	1.25 (0.89–1.76)
11–20 years	2281/178.6	1.14 (1.07–1.23)	1615/180.6	1.19 (1.01–1.41)
21– years	6503/449.0	1.09 (1.06–1.12)	4557/451.5	1.12 (1.06–1.18)
*P for trend*		0.27		0.001

^**a**^Adjusted for attained age, sex, calendar period at follow-up, and highest educational attainment; sibling comparison was also conditioned on family identifiers

**Table 4:** Incidence rate (IR, per 100,000 person-years) and hazard ratio (HR) with 95% confidence interval (CI) of any cancer in relation to surgical removal of tonsils and adenoids, stratified analysis by sex, age at follow-up, and educational attainment (3-year lag time)

	**Population comparison**			
**Characteristics**	**Individuals with surgery**		**Unexposed population reference**	
	***N*** **of cases/IR**	**HR (95%CI)**^**a**^	***N*** **of cases/IR**	**HR (95%CI)**^**a**^
**Sex**				
Male	3863/239.8	1.10 (1.06–1.13)	289,754/348.8	Ref
Female	5915/280.2	1.06 (1.03–1.09)	289,697/367.6	Ref
*P for difference*		0.06		
**Age at follow-up**				
< 20	52/21.3	1.28 (0.97–1.68)	2985/15.2	Ref
21–40	1452/79.6	1.08 (1.02–1.14)	38,207/66.8	Ref
41–	8274/500.2	1.08 (1.06–1.11)	538,259/632.8	Ref
*P for difference*		0.36		
**Educational attainment**				
–9 years	1870/377.0	1.11 (1.06–1.17)	176,912/545.5	Ref
> 9–12 years	4549/244.2	1.06 (1.03–1.09)	239,727/324.1	Ref
> 12 years	3271/240.7	1.11 (1.07–1.15)	149,981/273.4	Ref
Unknown	88/2131.9	1.46 (1.18–1.80)	12,831/2098.0	Ref
*P for difference*		0.05		
	**Sibling comparison**			
**Characteristics**	**Individuals with surgery**		**Unaffected siblings**	
	***N*** **of cases/IR**	**HR (95%CI)**^**b**^	***N*** **of cases/IR**	**HR (95%CI)**^**b**^
**Sex**				
Male	2700/240.3	1.16 (1.06–1.28)	8788/223.3	Ref
Female	4148/281.0	1.14 (1.06–1.23)	8947/233.2	Ref
*P for difference*		0.75		
**Age at follow-up**				
< 20	37/23.2	0.94 (0.51–1.72)	251/15.1	Ref
21–40	1010/80.0	1.16 (1.03–1.30)	2125/63.9	Ref
41–	5801/492.7	1.15 (1.09–1.20)	15,359/551.2	Ref
*P for difference*		0.78		
**Educational attainment**				
–9 years	1277/373.1	1.12 (0.97–1.28)	4749/378.4	Ref
> 9–12 years	3169/246.3	1.14 (1.04–1.23)	8085/212.9	Ref
> 12 years	2373/245.0	1.25 (1.13–1.39)	4773/176.1	Ref
Unknown	29/1618.8	Not estimable	128/1461.7	Ref
*P for difference*		0.85		

^a^Adjusted for attained age, sex, calendar period at follow-up, and educational attainment

^**b**^Conditioned on family identifiers and adjusted for attained age, sex, calendar period at follow-up, and educational attainment

When restricting the analysis to exposed and unexposed individuals with an indication for surgical removal of tonsils and adenoids, we still found a positive association for any cancer (HR 1.07; 95%CI 1.05–1.10). Excluding individuals that had the procedure before cohort entry did not change the results greatly (HR 1.11; 95%CI 1.08–1.14 for any cancer). Analysis by age at follow-up also rendered similar results as those in the main analysis (HR 1.21; 95%CI 0.86–1.70 for < 20; HR 1.12; 95%CI 1.05–1.19 for 21–40; and HR 1.11; 95%CI 1.07–1.15 for 41–). Including the first three years of follow-up in the exposed group did not change the results greatly either (HR 1.11; 95%CI 1.09–1.13 for any cancer).

## Discussion

Based on a population-based and sibling-controlled cohort study of almost five million individuals with a follow-up of up to 36 years, we found a surgical removal of tonsils and adenoids to be associated with a modestly increased overall risk of cancer during the decades following the surgery. Among individual cancer types, an increased risk was noted for breast cancer, prostate cancer, thyroid cancer, and lymphoma. These associations are corroborated between the population and sibling comparisons, suggesting that they are unlikely attributed to confounding due to shared genetic, lifestyle, or environmental factors within a family.

Given the widespread use of tonsillectomy, adenotonsillectomy, and adenoidectomy as well as the immune function of tonsils and adenoids, multiple studies have examined the relationship between these procedures and risk of cancer. In our literature review, we identified 42 studies that investigated risk of cancer in relation to tonsillectomy, among which 23 studied hematopoietic malignancies, 14 studied a specific type or group of solid tumors, whereas five studied the overall risk of cancer (Table 1). Among the five studies that examined the overall risk of cancer, one is a cohort study whereas the others are case–control studies. The cohort study and one case–control study found a positive association between tonsillectomy and risk of cancer, in agreement with our study [44, 47], whereas the other three reported either a null [46, 48] or an inverse [45] association. Although not statistically significant, one of the two null studies did also report a higher risk of any cancer in relation to tonsillectomy [48]. It is difficult to compare the results of the other two studies [45, 46] to that of the present study. For instance, it is unclear whether some, if any, of the cancer patients included in the studies were newly diagnosed as in the present study. Inclusion of prevalent cases could lead to selection bias as patients surviving a cancer might differ from newly diagnosed patients. The fact that the controls of the two studies consisted of residents of homes for elderly, hospital personnel, and patients with noncancerous diseases constitutes another concern of selection bias as it may indeed be unlikely that these controls adequately reflect the exposure prevalence in the source population that gave rise to the cases.

Our findings on breast cancer, prostate cancer, thyroid cancer, and lymphoma are partly in accordance with the existing literature. In our literature review (Table 1), we identified four original studies and one meta-analysis on breast cancer, which all showed a positive association between tonsillectomy and risk of breast cancer [36, 38, 43, 44, 51], either overall or in specific groups. We found two studies on prostate cancer [41, 52], with one reporting a positive association [41] and another reporting a null association [39]. The latter study was, however, small and likely underpowered. We found only one study on thyroid cancer without showing a positive association [53]. The study included however only 100 cases and 100 controls. Among studies on hematopoietic malignancies, many analyzed Hodgkin’s disease [8–10, 12, 14–29], including five studies reporting a positive association [8, 20, 21, 26, 28] and two studies reporting a non-significantly higher risk of Hodgkin’s disease following tonsillectomy [9, 10]. Two of these studies are cohort studies [8, 9], like the present study. However, all three studies have a relatively small number of incident cases with Hodgkin’s disease (73 or fewer among individuals exposed to tonsillectomy), making false positive or negative findings a concern in all. Other studies reported a null association, often based on a small sample size [12, 14, 16, 18, 23, 25, 29]. Another two studies examined non-Hodgkin lymphoma [10, 12] without reaching a consensus.

In addition to these main findings, which are consistent between the population and sibling comparisons, we also observed a higher risk of esophageal cancer, pancreatic cancer, kidney cancer, and leukemia following a surgical removal of tonsils and adenoids in either the population or sibling comparison. In line with our finding, the only prior study on esophageal cancer also reported a positive association [34]. In contrast, three previous studies on pancreatic cancer are at odds with our study, showing a null [40] or inverse [35, 42] association between tonsillectomy and risk of pancreatic cancer. We identified four studies on leukemia, with one cohort study demonstrating a positive association between tonsillectomy and acute leukemia in childhood [13] whereas the other three showing a null result [11, 12, 30]. Finally, we found four studies on head and neck cancer, with one cohort study showing a higher risk of head and neck cancer in relation to tonsillectomy among individuals at 60 and above but not younger [32], whereas others, including one cohort and two case–control studies, showed mixed results [2, 31, 33]. In the present study, a higher risk of head and neck cancer following a surgical removal of tonsils and adenoids was suggested in sibling comparison but not in population comparison. The lack of association in the population comparison corroborates with the null finding of one existing cohort study [31]. The positive association noted among individuals at 60 or above of the other cohort study [32] should also be interpreted with caution given the very small number of cases identified among individuals with tonsillectomy (23 or fewer).

An increased risk of cancer following surgical removal of tonsils and adenoids might be due to multiple reasons. Tonsils and adenoids, as secondary lymphoid organs, produce secondary immune responses through exhibiting specific antibodies and B and T cell activities, demonstrating humoral and cellular immune functions [54]. Removal of lymphoid tissues and its resultant immune-response alterations might therefore contribute to an increased risk of cancer [13, 55]. Alternatively, individuals receiving a surgical removal of tonsils and adenoids might have impaired immune responses to combat the underlying conditions (e.g., hypertrophic tonsils, chronic infection, and recurrent middle ear effusion), regardless of the surgical procedure. Although previous studies failed to demonstrate significant damages to the immune system [56–60] or showed only a transient decline in immunity [61] subsequent to tonsillectomy, multiple studies have indeed shown an increased risk of autoimmune disease [62], irritable bowel disease specifically [5], as well as a collective group of respiratory, allergic, and infectious diseases [7], demonstrating the presence of immune dysfunction among individuals with a previous tonsillectomy. Finally, the indications of the surgical removal, such as chronic infection, rather than the surgical procedure itself, might increase the risk of certain cancers. An infectious etiology is indeed confirmed for many cancer types, and estimated to be responsible for 10–20% of all human cancers [63]. The similar result on any cancer shown in sensitivity analysis comparing exposed individuals with registered indications for surgical removal of tonsils or adenoids to unexposed individuals with similar diagnoses argues against indication bias as the sole explanation for the observed association. This sensitivity analysis is however not free of bias. For instance, we identified potential indications through registered hospital diagnoses alone and did not have detailed clinical information. As a result, although both groups had diagnoses that could potentially serve as indications for the surgical removal of tonsils and adenoids, individuals that eventually underwent the surgical procedure might have had “real indications” (e.g., severe and persistent symptoms) whereas individuals that did not undergo the surgical procedure might not have had such “real indications” (e.g., milder and transient symptoms) or have had contraindications for the surgical removal of tonsils and adenoids (e.g., anemia, poor anesthetic risk, and acute infection). Such differences might be associated with the risk of cancer in general or the risk of specific cancers (e.g., hematopoietic malignancies), leading to biased results. Regardless, irrespective of the underlying reasons (most likely multifold), this finding is still of substantial public health importance, given the high prevalence of the procedure (11% in the present study population) and the persistently increased risk of cancer more than 20 years after the procedure.

Strengths of the present study include the population-based and sibling-controlled cohort design, the large sample size, the long and complete follow-up, and the prospective and independent collection of data on surgical procedures as well as cancer occurrence. The population-based design and the complete follow-up alleviated concerns on selection bias. The large sample size and long follow-up provided a unique opportunity to examine different characteristics of the exposure and individual types of cancer without a high risk of chance finding. The prospective and independent collection of data on exposure, outcome, and covariables allayed concerns on information bias often seen in cross-sectional studies (e.g., case–control studies). The possibility to cross-validate findings between population comparison and sibling comparison alleviated concerns on residual confounding due to factors potentially shared between siblings, genetic, lifestyle, or environmental. Finally, unlike previous studies that almost exclusively studied tonsillectomy, our study is the first effort to assess simultaneously tonsillectomy, adenotonsillectomy, and adenoidectomy and demonstrates a similar risk increase in cancer following all three procedures.

Limitations of the study include firstly that we did not have access to the medical records of the surgeries and had to rely on previous disease history when identifying potential indications for the surgical removal of tonsils and adenoids. Second, although we contrasted findings between population and sibling comparisons, the sibling comparison does not consider risk factors not shared between siblings. Finally, whether the findings of the present study could be readily generalizable to other settings with different healthcare systems is uncertain.

## Conclusions

Surgical removal of tonsils and adenoids is associated with a modestly increased risk of cancer during the decades following the surgery. The association is unlikely attributed to confounding due to shared genetic or non-genetic factors with a family.

## Supplementary Information

**Additional file 1: Table S1. **ICD codes for malignancies. **Table S2.** ICD codes for indications of surgical removal of tonsils and adenoids. **Table S3.** IR and HR of cancer in relation to surgical removal of tonsils and adenoids, population-based comparison. **Table S4.** IR and HR of cancer in relation to surgical removal of tonsils and adenoids, sibling comparison. **Table S5.** HR of cancer in relation to surgical removal of tonsils and adenoids, population-based comparison by sex.

## Data Availability

Data are from the Swedish population and health registers. According to the Swedish law, data cannot be put into a public data repository but are available by applying through Statistics Sweden or the Swedish National Board of Health and Welfare. Detailed information on data application can be found in their official sites: https://www.scb.se/vara-tjanster/bestall-data-och-statistik/bestalla-mikrodata/ and https://bestalladata.socialstyrelsen.se.

## Abbreviations

IR: Incidence rate
HR: Hazard ratio
CI: Confidence interval

## References

[1] Ramos SD, Mukerji S, Pine HS (2013). Tonsillectomy and adenoidectomy. Pediatr Clin North Am.

[2] Zevallos JP, Mazul AL, Rodriguez N, Weissler MC, Brennan P, Anantharaman D (2016). Previous tonsillectomy modifies odds of tonsil and base of tongue cancer. Br J Cancer.

[3] Mitchell RB, Archer SM, Ishman SL, Rosenfeld RM, Coles S, Finestone SA (2019). Clinical practice guideline: tonsillectomy in children (update)-executive summary. Otolaryngol Head Neck Surg.

[4] Kubba H, Downie LS (2021). Trends in tonsillectomy surgery in children in Scotland 2000–2018. Clin Otolaryngol.

[5] Bager P, Gørtz S, Feenstra B, Nyboe Andersen N, Jess T, Frisch M (2019). Increased Risk of Inflammatory Bowel Disease in Families with Tonsillectomy: A Danish National Cohort Study. Epidemiology.

[6] Janszky I, Mukamal KJ, Dalman C, Hammar N, Ahnve S (2011). Childhood appendectomy, tonsillectomy, and risk for premature acute myocardial infarction–a nationwide population-based cohort study. Eur Heart J.

[7] Byars SG, Stearns SC, Boomsma JJ (2018). Association of long-term risk of respiratory, allergic, and infectious diseases with removal of adenoids and tonsils in childhood. JAMA Otolaryngol Head Neck Surg.

[8] Vestergaard H, Westergaard T, Wohlfahrt J, Hjalgrim H, Melbye M (2010). Tonsillitis, tonsillectomy and Hodgkin's lymphoma. Int J Cancer.

[9] Liaw KL, Adami J, Gridley G, Nyren O, Linet MS (1997). Risk of Hodgkin's disease subsequent to tonsillectomy: a population-based cohort study in Sweden. Int J Cancer.

[10] Becker N, Deeg E, Rüdiger T, Nieters A (2005). Medical history and risk for lymphoma: results of a population-based case-control study in Germany. Eur J Cancer (Oxford, England: 1990)..

[11] Vineis P, Miligi L, Crosignani P, Davico L, Fontana A, Masala G (2003). Delayed infection, late tonsillectomy or adenoidectomy and adult leukaemia: a case-control study. Br J Cancer.

[12] Vineis P, Crosignani P, Sacerdote C, Fontana A, Masala G, Miligi L (2000). Haematopoietic cancer and medical history: a multicentre case control study. J Epidemiol Community Health.

[13] Schüz J, Kaletsch U, Meinert R, Kaatsch P, Michaelis J (1999). Association of childhood leukaemia with factors related to the immune system. Br J Cancer.

[14] Gledovic Z, Radovanovic Z (1991). History of tonsillectomy and appendectomy in Hodgkin's disease. Eur J Epidemiol.

[15] Serraino D, Franceschi S, Talamini R, Barra S, Negri E, Carbone A (1991). Socio-economic indicators, infectious diseases and Hodgkin's disease. Int J Cancer.

[16] Bonelli L, Vitale V, Bistolfi F, Landucci M, Bruzzi P (1990). Hodgkin's disease in adults: association with social factors and age at tonsillectomy. A case-control study. Int J Cancer.

[17] Mueller N, Swanson GM, Hsieh CC, Cole P (1987). Tonsillectomy and Hodgkin's disease: results from companion population-based studies. J Natl Cancer Inst.

[18] Hardell L, Bengtsson NO (1983). Epidemiological study of socioeconomic factors and clinical findings in Hodgkin's disease, and reanalysis of previous data regarding chemical exposure. Br J Cancer.

[19] Silingardi V, Venezia L, Tampieri A, Gramolini C (1982). Tonsillectomy, appendectomy and malignant lymphomas. Scand J Haematol.

[20] Vianna NJ, Lawrence CE, Davies JN, Arbuckle J, Harris S, Marani W (1980). Tonsillectomy and childhood Hodgkin's disease. Lancet (London, England).

[21] Kirchhoff LV, Evans AS, McClelland KE, Carvalho RP, Pannuti CS (1980). A case-control study of Hodgkin's disease in Brazil. I. Epidemiogic aspects. Am J Epidemiol.

[22] Henderson BE, Dworsky R, Pike MC, Baptista J, Menck H, Preston-Martin S (1979). Risk factors for nodular sclerosis and other types of Hodgkin's disease. Can Res.

[23] Andersen E, Isager H (1978). Pre-morbid factors in Hodgkin's disease. II. BCG-vaccination status, tuberculosis, infectious diseases, tonsillectomy, and appendectomy. Scand J Haematol.

[24] Abramson JH, Pridan H, Sacks MI, Avitzour M, Peritz E (1978). A case-control study of Hodgkin's disease in Israel. J Natl Cancer Inst.

[25] Paffenbarger RS, Wing AL, Hyde RT (1977). Characteristics in youth indicative of adult-onset Hodgkin's disease. J Natl Cancer Inst.

[26] Vianna NJ, Greenwald P, Polan A, Keogh MD, Davies JN (1974). Letter: Tonsillectomy and Hodgkin's disease. Lancet (London, England).

[27] Johnson SK, Johnson RE (1972). Tonsillectomy history in Hodgkin's disease. N Engl J Med.

[28] Vianna NJ, Greenwald P, Davies JN (1971). Tonsillectomy and Hodgkin's disease: the lymphoid tissue barrier. Lancet (London, England).

[29] Ruuskanen C, Vanha-Perttula T, Kouvalainen K (1971). Tonsillectomy, appendicectomy, and Hodgkin's disease. Lancet (London, England).

[30] Freeman AI, Lieberman N, Tidings J, Bross I, Glidewell O (1971). Previous tonsillectomy and the incidence of acute leukaemia of childhood. Lancet (London, England).

[31] Chaturvedi AK, Song H, Rosenberg PS, Ramqvist T, Anderson WF, Munck-Wikland E (2016). Tonsillectomy and incidence of oropharyngeal cancers. Cancer Epidemiol Biomarkers Prev.

[32] Fakhry C, Andersen KK, Christensen J, Agrawal N, Eisele DW (2015). The impact of tonsillectomy upon the risk of oropharyngeal carcinoma diagnosis and prognosis in the Danish cancer registry. Cancer Prev Res (Phila).

[33] Combes JD, Voisin N, Périé S, Malard O, Jegoux F, Nadjingar R (2021). History of tonsillectomy and risk of oropharyngeal cancer. Oral Oncol.

[34] Garman KS, Ajayi TA, Boutte HJ, Chiu ST, von Furstenberg RJ, Lloyd BR (2020). Prior tonsillectomy is associated with an increased risk of esophageal adenocarcinoma. PLoS One.

[35] Zhang J, Prizment AE, Dhakal IB, Anderson KE (2014). Cholecystectomy, gallstones, tonsillectomy, and pancreatic cancer risk: a population-based case-control study in Minnesota. Br J Cancer.

[36] Brasky TM, Bonner MR, Dorn J, Marhsall JR, Vena JE, Brasure JR (2009). Tonsillectomy and breast cancer risk in the Western New York diet study. Cancer Causes Control.

[37] Zivaljevic V, Vlajinac H, Jankovic R, Marinkovic J, Diklic A, Paunovic I (2004). Case-control study of anaplastic thyroid cancer. Tumori.

[38] Yasui Y, Potter JD, Stanford JL, Rossing MA, Winget MD, Bronner M (2001). Breast cancer risk and "delayed" primary Epstein-Barr virus infection. Cancer Epidemiol Biomarkers Prev.

[39] Ilić M, Vlajinac H, Marinković J (1996). Case-control study of risk factors for prostate cancer. Br J Cancer.

[40] Bueno de Mesquita HB, Maisonneuve P, Moerman CJ, Walker AM (1992). Aspects of medical history and exocrine carcinoma of the pancreas: a population-based case-control study in The Netherlands. Int J Cancer.

[41] Whittemore AS, Paffenbarger RS, Anderson K, Lee JE (1985). Early precursors of site-specific cancers in college men and women. J Natl Cancer Inst.

[42] Gold EB, Gordis L, Diener MD, Seltser R, Boitnott JK, Bynum TE (1985). Diet and other risk factors for cancer of the pancreas. Cancer.

[43] Lubin JH, Burns PE, Blot WJ, Lees AW, May C, Morris LE (1982). Risk factors for breast cancer in women in northern Alberta, Canada, as related to age at diagnosis. J Natl Cancer Inst.

[44] Sun LM, Chen HJ, Li TC, Sung FC, Kao CH (2015). A nationwide population-based cohort study on tonsillectomy and subsequent cancer incidence. Laryngoscope.

[45] Cassimos C, Sklavunu-Zurukzoglu S, Catriu D, Panajiotidu C (1973). The frequency of tonsillectomy and appendectomy in cancer patients. Cancer.

[46] Gross L (1966). Incidence of appendectomies and tonsillectomies in cancer patients. Cancer.

[47] Howie JG, Timperley WR (1966). Cancer and appendectomy. Cancer.

[48] Kessler II (1970). Lymphoid tissues in neoplasia. A pilot study and review. Cancer.

[49] Barlow L, Westergren K, Holmberg L, Talbäck M (2009). The completeness of the Swedish Cancer Register: a sample survey for year 1998. Acta oncologica (Stockholm, Sweden).

[50] Ludvigsson JF, Andersson E, Ekbom A, Feychting M, Kim JL, Reuterwall C (2011). External review and validation of the Swedish national inpatient register. BMC Public Health.

[51] Kacimi SEO, Elgenidy A, Cheema HA, Ould Setti M, Khosla AA, Benmelouka AY (2022). Prior tonsillectomy and the risk of breast cancer in females: a systematic review and meta-analysis. Front Oncol.

[52] Whittemore AS, Paffenbarger RS, Anderson K, Lee JE (1984). Early precursors of urogenital cancers in former college men. J Urol.

[53] Nagano J, Mabuchi K, Yoshimoto Y, Hayashi Y, Tsuda N, Land C (2007). A case-control study in Hiroshima and Nagasaki examining non-radiation risk factors for thyroid cancer. J Epidemiol.

[54] Brandtzaeg P (2011). Potential of nasopharynx-associated lymphoid tissue for vaccine responses in the airways. Am J Respir Crit Care Med.

[55] Holló G (2021). Tonsillectomy and the incidence of various types of cancer. Immunol Res.

[56] Ikincioğullari A, Doğu F, Ikincioğullari A, Eğin Y, Babacan E (2002). Is immune system influenced by adenotonsillectomy in children?. Int J Pediatr Otorhinolaryngol.

[57] Nasrin M, Miah MR, Datta PG, Saleh AA, Anwar S, Saha KL (2012). Effect of tonsillectomy on humoral immunity. Bangladesh Med Res Counc Bull.

[58] Böck A, Popp W, Herkner KR (1994). Tonsillectomy and the immune system: a long-term follow up comparison between tonsillectomized and non-tonsillectomized children. Eur Arch Otorhinolaryngol.

[59] Lal H, Sachdeva OP, Mehta HR (1984). Serum immunoglobulins in patients with chronic tonsillitis. J Laryngol Otol.

[60] Prusek W, Agopsowicz T, Podwysocka M (1983). T and B lymphocytes in peripheral blood and tonsils of children after tonsillectomy. Arch Immunol Ther Exp.

[61] Kaygusuz I, Gödekmerdan A, Karlidag T, Keleş E, Yalçin S, Aral I (2003). Early stage impacts of tonsillectomy on immune functions of children. Int J Pediatr Otorhinolaryngol.

[62] Ji J, Sundquist J, Sundquist K (2016). Tonsillectomy associated with an increased risk of autoimmune diseases: a national cohort study. J Autoimmun.

[63] de Martel C, Georges D, Bray F, Ferlay J, Clifford GM (2020). Global burden of cancer attributable to infections in 2018: a worldwide incidence analysis. Lancet Glob Health.

