# Supplementary material for: Cancer risk following surgical removal of tonsils and adenoids — a population-based, sibling-controlled cohort study in Sweden
Source: BMC Med. 2023 May 24;21:194. doi: 10.1186/s12916-023-02902-x (PMC10210283; doi:10.1186/s12916-023-02902-x)
Supplement: Supplementary file 1 — Additional file 1: Table S1. ICD codes for malignancies. Table S2. ICD codes for indications of surgical removal of tonsils and adenoids. Table S3. IR and HR of cancer in relation to surgical removal of tonsils and adenoids, population-based comparison. Table S4. IR and HR of cancer in relation to surgical removal of tonsils and adenoids, sibling comparison. Table S5. HR of cancer in relation to surgical removal of tonsils and adenoids, population-based comparison by sex. [file 12916_2023_2902_MOESM1_ESM.docx]

| **Table S1**: The 7^th^ Swedish revision of the International Classification of Diseases (ICD) codes used to identify different malignancies | |
| --- | --- |
| **Type of malignancies** | **ICD-7** |
| Any cancer | 140-209 |
| Head and neck cancer | 140-148 |
| Esophageal cancer | 150 |
| Stomach cancer | 151 |
| Small Intestine cancer including duodenum | 152 |
| Large intestine cancer | 153 |
| Rectum cancer | 154 |
| Primary liver Cancer | 155 |
| Pancreatic Cancer | 157 |
| Lung cancer | 161-163 |
| Melanoma skin cancer | 190 |
| Non-melanoma skin cancer | 191 |
| Breast cancer | 170 |
| Testis cancer | 178 |
| Cervix uteri cancer | 171 |
| Corpus uteri cancer | 172 |
| All uterus cancer | 171-174 |
| Ovarian cancer | 175 |
| Prostate cancer | 177 |
| Testis cancer | 178 |
| Bladder cancer | 181 |
| Kidney cancer | 180 |
| Central nervous system cancer | 193 |
| Cancer with non-specific sites | 199 |
| Thyroid cancer | 194 |
| Other cancer in the endocrine system | 195 |
| Connective tissue cancer | 197 |
| Lymphoma | Before 1975: 200, 201, 202, 205  1975 onward: 200, 201, 202 |
| Hodgkin's disease | 201 |
| Non-Hodgkin’s lymphoma | Before 1975: 200, 202  1975 onward: 200, 202 (exl. 2022) |
| Leukemia | 204-206 |

| **Table S2:** International Classification of Diseases (ICD) codes used to identify the indications for surgical removal of tonsils and adenoids | | | |
| --- | --- | --- | --- |
| **Groups** | **ICD-8** | **ICD-9** | **ICD-10** |
| 1. **Hypertrophy of tonsils and adenoids** | 500 | 474B | J35.1, J35.2, J35.3 |
| 1. **Other diseases of tonsils and adenoids** |  | 474C, 474W, 474X | J35.8, J35.9 |
| 1. **Chronic tonsilitis, pharyngitis and nasopharyngitis, or peritonsillar abscess** | 501, 502 | 472B, 473C, 474A, 475X | J31.1, J31.2, J35.0, J36 |
| 1. **Sleep disorders or dyspnea and respiratory abnormalities** | 306.4, 783.2, 783.6 | 780F, 786A, 786B | G47, R06.0, R06.1, R06.3, R06.5 |

| **Table S3.** Incidence rate (IR, per 100,000 person-years) and hazard ratio (HR) with 95% confidence interval (CI) of cancer in relation to surgical removal of tonsils and adenoids, population-based comparison (3-year lag time) | | | | |
| --- | --- | --- | --- | --- |
| **Type of malignancy** | **Individuals with surgery** | | **Unexposed population reference** | |
|  | **N of cases/IR** | **HR (95% CI)*** | **N of cases/IR** | **HR (95% CI)*** |
| Any cancer | 9,778/262.7 | 1.10 (1.07-1.12) | 579,451/357.9 | Ref |
| Head and neck | 183/4.9 | 1.02 (0.88-1.19) | 11,240/6.9 | Ref |
| Esophagus | 61/1.6 | 1.26 (0.98-1.63) | 4,236/2.6 | Ref |
| Stomach | 108/2.9 | 1.16 (0.96-1.40) | 7,799/4.8 | Ref |
| Small intestine | 50/1.3 | 1.24 (0.93-1.64) | 2,661/1.6 | Ref |
| Large intestine | 447/12.0 | 1.02 (0.93-1.12) | 33,706/20.8 | Ref |
| Rectum | 285/7.6 | 1.00 (0.89-1.13) | 21,168/13.1 | Ref |
| Liver (primary) | 111/3.0 | 1.06 (0.88-1.28) | 7,980/4.9 | Ref |
| Pancreas | 155/4.2 | 1.23 (1.05-1.44) | 10,290/6.4 | Ref |
| Lung | 464/12.5 | 1.09 (1.00-1.20) | 37,024/22.9 | Ref |
| Breast | 2,153/102.0 | 1.06 (1.01-1.10) | 98,108/124.5 | Ref |
| Uterus | 510/24.2 | 0.98 (0.90-1.07) | 26,636/33.8 | Ref |
| Ovary | 193/9.1 | 0.91 (0.79-1.05) | 12,153/15.4 | Ref |
| Prostate | 1,226/76.1 | 1.15 (1.09-1.22) | 98,768/118.9 | Ref |
| Testis | 182/11.3 | 1.13 (0.98-1.31) | 6,095/7.3 | Ref |
| Kidney | 228/6.1 | 1.33 (1.16-1.52) | 12,962/8.0 | Ref |
| Bladder | 264/7.1 | 1.10 (0.97-1.24) | 21,663/13.4 | Ref |
| Malignant melanoma | 816/21.9 | 1.04 (0.97-1.12) | 36,418/22.5 | Ref |
| Non-melanoma skin | 286/7.7 | 1.14 (1.01-1.29) | 20,970/13.0 | Ref |
| Brain | 456/12.2 | 1.06 (0.96-1.16) | 21,905/13.5 | Ref |
| Thyroid | 163/4.4 | 1.18 (1.00-1.38) | 5,453/3.4 | Ref |
| Other endocrine system | 303/8.1 | 1.15 (1.02-1.29) | 12,627/7.8 | Ref |
| Connective tissue | 79/2.1 | 1.18 (0.94-1.48) | 3,686/2.3 | Ref |
| Non-specific sites | 151/4.0 | 0.98 (0.83-1.15) | 11,984/7.4 | Ref |
| Lymphoma | 384/10.3 | 1.11 (1.00-1.23) | 21,330/13.2 | Ref |
| Hodgkin lymphoma | 73/1.96 | 0.97 (0.77-1.22) | 3,167/1.96 | Ref |
| Non-Hodgkin lymphoma | 307/8.2 | 1.15 (1.03-1.29) | 17,873/11.0 | Ref |
| Leukemia | 296/8.0 | 1.22 (1.08-1.37) | 16,150/10.0 | Ref |
| ^*^Adjusted for sex, age and calendar period at follow-up, and educational attainment. | | | | |

| **Table S4.** Incidence rate (IR, per 100,000 person-years) and hazard ratio (HR) with 95% confidence interval (CI) of cancer in relation to surgical removal of tonsils and adenoids, sibling comparison (3-year lag time) | | | | |
| --- | --- | --- | --- | --- |
| **Type of malignancy** | **Individuals with surgery** | | **Unaffected siblings** | |
|  | **N of cases/IR** | **HR (95% CI)*** | **N of cases/IR** | **HR (95% CI)*** |
| Any cancer | 6,848/263.4 | 1.15 (1.10-1.20) | 17,735/228.2 | Ref |
| Head and neck | 122/4.7 | 1.36 (0.97-1.90) | 1,164/15.0 | Ref |
| Esophagus | 37/1.4 | 2.09 (1.02-4.31) | 82/1.0 | Ref |
| Stomach | 71/2.7 | 1.07 (0.68-1.67) | 173/2.2 | Ref |
| Small intestine | 35/1.3 | 1.26 (0.65-2.47) | 64/0.8 | Ref |
| Large intestine | 324/12.5 | 1.22 (0.97-1.52) | 793/10.2 | Ref |
| Rectum | 186/7.2 | 1.05 (0.82-1.36) | 485/6.2 | Ref |
| Liver (primary) | 81/3.1 | 1.45 (0.91-2.31) | 201/2.6 | Ref |
| Pancreas | 109/4.2 | 1.23 (0.86-1.75) | 237/3.0 | Ref |
| Lung | 302/11.6 | 1.06 (0.86-1.31) | 912/11.7 | Ref |
| Breast | 1,534 /103.9 | 1.16 (1.02-1.32) | 2,905/75.7 | Ref |
| Uterus | 343/23.2 | 0.98 (0.76-1.27) | 768/20.0 | Ref |
| Corpus uteri | 140/9.5 | 1.22 (0.81-1.84) | 339/8.8 | Ref |
| Cervical cancer | 188/12.7 | 0.95 (0.67-1.33) | 384/10.0 | Ref |
| Ovary | 137/9.3 | 0.94 (0.64-1.39) | 337/8.8 | Ref |
| Prostate | 882/78.5 | 1.24 (1.05-1.48) | 2,392/60.8 | Ref |
| Testis | 132/11.7 | 0.92 (0.61-1.39) | 323/8.2 | Ref |
| Kidney | 156/6.0 | 1.30 (0.94-1.81) | 385/5.0 | Ref |
| Bladder | 177/6.8 | 1.29 (0.95-1.76) | 486/6.2 | Ref |
| Malignant melanoma | 579/22.3 | 1.09 (0.94-1.26) | 1,252/16.1 | Ref |
| Non-melanoma skin | 195/7.5 | 0.91 (0.69-1.21) | 473/6.1 | Ref |
| Brain | 328/12.6 | 1.10 (0.90-1.35) | 807/10.4 | Ref |
| Thyroid | 116/4.5 | 1.63 (1.11-2.40) | 246/3.2 | Ref |
| Other endocrine system | 217/8.3 | 1.09 (0.85-1.40) | 499/6.4 | Ref |
| Connective tissue | 58/2.2 | 1.14 (0.70-1.87) | 133/1.7 | Ref |
| Non-specific sites | 100/3.8 | 0.88 (0.63-1.22) | 592/7.6 | Ref |
| Lymphoma | 279/10.7 | 1.28 (1.03-1.61) | 990/12.7 | Ref |
| Hodgkin lymphoma | 57/2.2 | 1.35 (0.85-2.14) | 185/2.4 | Ref |
| Non-Hodgkin lymphoma | 219/8.4 | 1.26 (0.97-1.63) | 799/10.3 | Ref |
| Leukemia | 202/7.8 | 1.23 (0.94-1.60) | 562/7.2 | Ref |
| ^*^Conditioned on family identifiers and adjusted for sex, age and calendar period at follow-up, and educational attainment. | | | | |

| **Table S5.** Hazard ratio (HR) with 95% confidence interval (CI) of cancer in relation to surgical removal of tonsils and adenoids, population-based comparison by sex (3-year lag time) | | | | |
| --- | --- | --- | --- | --- |
| **Type of malignancy** | **Male** | | **Female** | |
|  | **N of cases/IR** | **HR (95% CI)*** | **N of cases/IR** | **HR (95% CI)*** |
| Any cancer | 3863/239.8 | 1.10 (1.06-1.13) | 5915/280.2 | 1.06 (1.03-1.09) |
| Head and neck | 105/6.5 | 1.02 (0.84-1.24) | 78/3.7 | 1.01 (0.80-1.26) |
| Esophagus | 43/2.7 | 1.20 (0.89-1.60) | 18/0.8 | 1.44 (0.90-2.30) |
| Stomach | 58/3.6 | 1.12 (0.86-1.45) | 50/2.4 | 1.20 (0.90-1.58) |
| Small intestine | 22/1.4 | 1.08 (0.71-1.65) | 28/1.3 | 1.40 (0.96-2.04) |
| Large intestine | 199/12.4 | 1.03 (0.89-1.18) | 248/11.7 | 1.01 (0.89-1.14) |
| Rectum | 127/7.9 | 0.89 (0.75-1.06) | 158/7.5 | 1.09 (0.93-1.28) |
| Liver (primary) | 60/3.7 | 1.14 (0.88-1.47) | 51/2.4 | 1.02 (0.77-1.34) |
| Pancreas | 70/4.3 | 1.22 (0.96-1.55) | 85/4.0 | 1.24 (1.00-1.54) |
| Lung | 162/10.0 | 0.94 (0.81-1.10) | 302/14.3 | 1.16 (1.04-1.30) |
| Breast | - | - | 2,153/102.0 | 1.06 (1.01-1.10) |
| Uterus | 510/24.2 | 0.98 (0.90-1.07) | 26,636/33.8 | Ref |
| Ovary | 193/9.1 | 0.91 (0.79-1.05) | 12,153/15.4 | Ref |
| Prostate | 1,226/76.1 | 1.15 (1.09-1.22) | - | - |
| Testis | 182/11.3 | 1.13 (0.98-1.31) | - | - |
| Kidney | 126/7.8 | 1.27 (1.06-1.52) | 102/4.8 | 1.43 (1.17-1.74) |
| Bladder | 172/10.7 | 1.05 (0.90-1.22) | 92/4.4 | 1.18 (0.96-1.46) |
| Malignant melanoma | 816/21.9 | 1.04 (0.97-1.12) | 36,418/22.5 | 1.09 (0.97-1.23) |
| Non-melanoma skin | 137/8.5 | 1.14 (0.96-1.35) | 149/7.0 | 1.11 (0.94-1.31) |
| Brain | 174/10.8 | 1.00 (0.86-1.17) | 282/13.4 | 1.09 (0.97-1.23) |
| Thyroid | 37/2.3 | 1.33 (0.96-1.85) | 126/6.0 | 1.12 (0.94-1.34) |
| Other endocrine system | 89/5.5 | 1.13 (0.92-1.40) | 214/10.1 | 1.15 (1.00-1.32) |
| Connective tissue | 42/2.6 | 1.30 (0.96-1.77) | 37/1.8 | 1.07 (0.77-1.49) |
| Non-specific sites | 51/3.2 | 0.83 (0.63-1.10) | 100/4.7 | 1.07 (0.88-1.31) |
| Lymphoma | 227/14.1 | 1.27 (1.11-1.45) | 157/7.4 | 0.94 (0.80-1.10) |
| Hodgkin lymphoma | 39/2.4 | 1.07 (0.78-1.47) | 34/1.6 | 0.87 (0.62-1.22) |
| Non-Hodgkin lymphoma | 185/11.5 | 1.32 (1.14-1.53) | 122/5.8 | 0.97 (0.81-1.16) |
| Leukemia | 140/8.7 | 1.17 (0.99-1.38) | 156/7.4 | 1.26 (1.07-1.48) |
| ^*^Adjusted for age and calendar period at follow-up, and educational attainment. | | | | |
